# Metabolic syndrome in patients with first-ever ischemic stroke: prevalence and association with coronary heart disease

**DOI:** 10.1038/s41598-022-17369-8

**Published:** 2022-07-29

**Authors:** Yajun Liang, Zhongrui Yan, Yanlei Hao, Qiqi Wang, Zuoji Zhang, Rui She, Peng Wang, Yifeng Du, Joseph TF Lau, Joost Dekker, Bo Bai, Chengxuan Qiu

**Affiliations:** 1 Aging Research Center & Center for Alzheimer Research, Department of Neurobiology, Care Sciences and Society, Karolinska Institutet-Stockholm University, Tomtebodavägen 18A, 17165 Stockholm, Sweden; 2 Department of Global Public Health, Karolinska Institutet, Stockholm, Sweden; 3 Department of Neurology, Jining No. 1 People’s Hospital, Jining, Shandong China; 4 The Affiliated Hospital of Jining Medical University, Jining, Shandong China; 5 Office of Epidemiology, Chinese Center for Disease Control and Prevention, Beijing, China; 6 Jining Medical University, Hehua Road 133, Taibaihu New District, Jining, 272067 Shandong China; 7 JC School of Public Health and Primary Care, The Chinese University of Hong Kong, Hong Kong, China; 8 Department of Neurology, Shandong Provincial Hospital Affiliated to Shandong University, Jinan, Shandong China; 9 Department of Psychiatry and Department of Rehabilitation Medicine, Amsterdam University Medical Centres, Amsterdam, The Netherlands

**Keywords:** Cardiology, Medical research

## Abstract

The metabolic syndrome (MetS) has been well linked with coronary heart disease (CHD) in the general population, but studies have rarely explored their association among patients with stroke. We examine prevalence of MetS and its association with CHD in patients with first-ever ischemic stroke. This hospital-based study included 1851 patients with first-ever ischemic stroke (mean age 61.2 years, 36.5% women) who were hospitalized into two university hospitals in Shandong, China (January 2016–February 2017). Data were collected through interviews, physical examinations, and laboratory tests. MetS was defined following the National Cholesterol Education Program (NCEP) criteria, the International Diabetes Federation (IDF) criteria, and the Chinese Diabetes Society (CDS) criteria. CHD was defined following clinical criteria. Data were analyzed using binary logistic regression models. The overall prevalence of MetS was 33.4% by NECP criteria, 47.2% by IDF criteria, and 32.5% by CDS criteria, with the prevalence being decreased with age and higher in women than in men (p < 0.05). High blood pressure, high triglycerides, and low HDL-C were significantly associated with CHD (multi-adjusted odds ratio [OR] range 1.27–1.38, p < 0.05). The multi-adjusted OR of CHD associated with MetS defined by the NECP criteria, IDF criteria, and CDS criteria (vs. no MetS) was 1.27 (95% confidence interval 1.03–1.57), 1.44 (1.18–1.76), and 1.27 (1.03–1.57), respectively. In addition, having 1–2 abnormal components (vs. none) of MetS was associated with CHD (multi-adjusted OR range 1.66–1.72, p < 0.05). MetS affects over one-third of patients with first-ever ischemic stroke. MetS is associated with an increased likelihood of CHD in stroke patients.

## Introduction

The metabolic syndrome (MetS), characterized by a constellation of multiple interrelated cardiometabolic risk factors, has become a major concern for public health^1,2^. Currently, several criteria are proposed to define MetS such as the US National Cholesterol Education Program (NCEP) criteria^3^, the International Diabetes Federation (IDF) criteria^4^, and the Chinese Diabetes Society (CDS) criteria^5^. Thus, the prevalence of MetS varies across studies, and even in the same population, depending on the defining criteria^6^.

The associations between MetS and cardiovascular diseases have been well studied in the general population. A systematic review and meta-analysis of 87 population-based prospective studies showed that MetS was associated with an increased risk of cardiovascular disease (myocardial infarction, stroke) and cardiovascular mortality^7^. We previously reported that MetS was associated with coronary heart disease (CHD), stroke, and cardiovascular multimorbidity among Chinese older adults living in a rural area^6^. So far, data are sparse with regard to the relationship between MetS and CHD among patients with ischemic stroke.

Ischemic stroke and CHD are common circulatory disorders among adults and share major common etiological factors (e.g., smoking, hypertension, diabetes, and high cholesterol) and pathophysiological mechanisms (e.g., atherosclerosis)^8,9^. However, evidence also suggests that the two entities show differences in risk factors, pathophysiologies, incidence, mortality, and prognosis in the general population^10–12^. As the worldwide leading causes of disability and death, CHD and ischemic stroke together have a great impact on public health^13,14^. A meta-analysis suggested that coronary stenosis was highly prevalent in patients with ischemic stroke and that CHD was the leading cause of death following the occurrence of acute ischemic stroke^15^. A large-scale register-based study in Sweden showed that ~ 50% of the men with both stroke and coronary disease died from coronary heart disease (e.g., myocardial infarction and sudden coronary death)^10^. Similarly, a recent large-scale retrospective cohort study also revealed the poor prognosis and an increased risk of cardiovascular complications following the onset of an ischemic stroke^16^. Thus, identifying risk factors for CHD among stroke patients is crucial to reduce the risk of coronary events and improve the prognosis.

In this hospital-based study of patients with first-ever acute ischemic stroke, we seek to describe the prevalence of MetS and CHD, and further to assess the association of MetS with CHD among the patients with ischemic stroke.

## Methods

### Study design and population

Data were obtained from the baseline survey of a hospital-based intervention study, the Multimodal Behavioral Intervention Study in Stroke, which is an ongoing randomized controlled multimodal intervention study in two hospitals, i.e., the Shandong Jining No. 1 People’s Hospital and the Jining Medical University Affiliated Hospital, Shandong, China^17^. The recruitment and baseline survey of participants was conducted from January 2016 to February 2017. In total, 2205 patients with first-ever acute ischemic stroke who were hospitalized into the above two hospitals were recruited based on the inclusion criteria similar to those specified in the China National Stroke Registry Protocol^18^: (a) first-ever ischemic stroke or transient ischemic attack (TIA); (b) age ≥ 40 years; (c) patients, family or caregivers can provide consent; (d) others (e.g., direct admission based on physician evaluation or arrival through the emergency department, and confirmed by brain CT or MRI within 14 days after the onset of symptoms). Of the 2205 participants, we excluded 354 patients who had insufficient information to define MetS, leaving 1851 patients (83.9%) for the current analysis.

### Data collection

Following the structured questionnaire, data were collected through interviews, clinical and neurological examinations, and laboratory tests by trained nurses, physicians, and technicians from the two hospitals, as previously reported^19^. Epidemiological data were collected via a questionnaire that was developed from the WHO STEPwise approach to Surveillance (STEPS) and the Study on Global Ageing and Adult Health (SAGE)^20,21^.

### MetS and its components

Waist circumference was measured at a point midway between the lowest rib and the iliac crest in a horizontal plane using nonelastic tape. After at least a 5-min rest, arterial blood pressure was measured in the sitting position on the right arm using an electronic sphygmomanometer (HEM-7127J, Omron Corporation, Kyoto, Japan) with the cuff maintained at the heart level. Blood pressure was measured three times on one occasion, and the mean of the three readings was used in the analysis. After an overnight fast, peripheral blood samples were taken at the hospital. Fasting blood glucose, triglycerides, and high-density lipoprotein cholesterol (HDL-C) were measured using an automatic Biochemical Analyser (Olympus AU-400, Olympus Optical Company, Tokyo, Japan) at the laboratory of the hospitals that is licensed by the local authority.

MetS were defined according to three sets of criteria: the NCEP criteria^3^, the IDF criteria^4^, and the CDS criteria^5^ (Table 1).

**Table 1:** Three sets of defining criteria for the metabolic syndrome.

Traits	NCEP criteria (at least three traits)^3^	IDF criteria (at least three traits)^4^	CDS criteria (at least three traits)^5^
Waist circumference	Men ≥ 102 cm; women ≥ 88 cm	Chinese men ≥ 85 cm; Chinese women ≥ 80 cm	Chinese men ≥ 90 cm; Chinese women ≥ 85 cm
Blood pressure	≥ 130/85 mmHg or use of antihypertensive drugs	≥ 130/85 mmHg or use of antihypertensive drugs	≥ 130/85 mmHg or use of antihypertensive drugs
Fasting plasma glucose	≥ 6.1 mmol/L or use of antidiabetic drugs	≥ 5.6 mmol/L or use of antidiabetic drugs	≥ 6.1 mmol/L or use of antidiabetic drugs
Serum triglycerides	≥ 1.7 mmol/L or use of lipid-lowering drugs	≥ 1.7 mmol/L or use of lipid-lowering drugs	≥ 1.7 mmol/L or use of lipid-lowering drugs
High-density lipoprotein cholesterol	Men < 1.04 mmol/L, women < 1.29 mmol/L or use of lipid-lowering drugs	Men < 1.04 mmol/L, women < 1.29 mmol/L or use of lipid-lowering drugs	< 1.04 mmol/L or lipid-lowering drugs

*NCEP* National Cholesterol Education Program, *IDF* International Diabetes Federation, *CDS* Chinese Diabetes Society.

### Definition of CHD

CHD was defined as coronary artery stenosis or occlusion caused by atherosclerosis in patients with a clear history of acute coronary syndrome or confirmed by coronary CT angiography or coronary angiography. The discharge diagnosis of CHD was made by senior cardiologists and neurologists via reviewing all medical records from comprehensive assessments during the hospitalization, which was based on medical history of CHD, clinical examinations, and instrumental assessments (e.g., coronary CT angiography or coronary angiography) following the current clinical guidelines^22^.

### Covariates

The covariates included age, sex, education, and lifestyles (e.g., smoking, alcohol drinking, physical activity, and dietary). Education was categorized into 4 groups: illiteracy (no formal schooling education), primary school (1–6 years of education), middle school (7–9 years of education), and high school and above (≥ 10 years of education). Smoking status was categorized as no smoking and ever smoking. Alcohol consumption was defined as drinking alcohol more than once per month during the past year. Physical inactivity was defined as having not participated in any physical activity during leisure time. Information on dietary habits was collected on the frequency of vegetables or fruits and categorized into daily versus less than daily consumption.

### Statistical analysis

The characteristics of study participants were compared between men and women with Student t-test for continuous variables and chi-square test for categorical variables. Due to the skewed distribution, triglycerides were logarithmized before the comparison between men and women. Because around 73.3% of the participants had missing values on waist circumference, a linear regression model (R^2^ = 17%, p < 0.001) was used to predict and impute the waist circumference based on body mass index and demographic data, as previously reported^23^. The age- and sex-specific prevalence was graphed for MetS and CHD. Binary logistic regression analysis was performed to estimate the odds ratio (OR) and 95% confidence interval (CI) of CHD associated with MetS and its components while adjusting for age, sex, education, smoking, alcohol drinking, physical activity, and dietary habits.

The IBM SPSS Statistics 25 for Windows (IBM SPSS Inc., Chicago, Illinois, USA) was used for all analyses.

### Ethics approval and consent to participate

The study protocols were approved by the Ethics Committee at Jining Medical University, Shandong, China (No. 2015B006). Written informed consent was obtained from all participants, or in case of cognitively impaired persons, from informants, usually the next-of-kin (spouse or children). Research within this project had been conducted according to the principles expressed in the Declaration of Helsinki.

## Results

The mean age of the 1851 participants was 61.2 (SD 9.7) years and 36.5% were women. Compared with men, women were older, less educated, and less likely to smoke, drink alcohol, and had higher levels of waist circumference, systolic blood pressure, fasting blood glucose, triglycerides, and HDL-C, had a lower level of diastolic blood pressure (all p < 0.01) (Table 2). There was no significant sex difference in the prevalence of physical inactivity and daily eating fruits and vegetables (p > 0.10).

**Table 2:** Characteristics of study participants by sex.

Characteristics^a^	Total (n = 1851)	Men (n = 1176)	Women (n = 675)	*P*
Age (years), mean (SD)	61.2 (9.7)	60.1 (9.5)	63.1 (9.7)	< 0.001
**Education, n (%)**				< 0.001
Illiteracy	476 (26.7)	125 (11.1)	351 (53.8)	
Primary school	463 (26.0)	306 (27.1)	157 (24.0)	
Middle school	501 (28.1)	398 (35.2)	103 (15.8)	
High school and above	343 (19.2)	301 (26.6)	42 (6.4)	
Ever smoking, n (%)	841 (45.4)	795 (67.6)	46 (6.8)	< 0.001
Alcohol drinking, n (%)	611 (35.6)	594 (56.8)	17 (2.5)	< 0.001
Physical inactivity, n (%)	533 (28.9)	326 (27.8)	207 (30.8)	0.176
Daily eating fruits and vegetables, n (%)	1659 (90.3)	1049 (89.8)	610 (91.0)	0.391
Waist circumference (cm), mean (SD)	81.7 (6.7)	82.4 (6.6)	80.6 (6.6)	< 0.001
Systolic blood pressure (mmHg), mean (SD)	140.8 (16.6)	139.7 (16.9)	142.9 (16.0)	< 0.001
Diastolic blood pressure (mmHg), mean (SD)	81.7 (11.3)	82.6 (11.5)	80.1 (10.8)	< 0.001
Blood glucose (mmol/l), mean (SD)	6.1 (2.3)	5.9 (2.2)	6.4 (2.5)	< 0.001
Serum triglycerides (mmol/l), median (IQR)	1.3 (0.9–1.8)	1.2 (0.9–1.8)	1.4 (1.0–1.9)	0.004
HDL-C (mmol/l), mean (SD)	1.2 (0.4)	1.1 (0.4)	1.2 (0.4)	< 0.001

*SD* standard deviation, *IQR* interquartile range, *HDL-C* high-density lipoprotein cholesterol.

^a^The number of missing values was 68 for education, 135 for alcohol drinking, 5 for physical activity, 13 for diet, 51 for waist circumference, 27 for blood pressure, 26 for blood glucose, 34 for serum triglycerides, and 51 for HDL-C.

**Figure 1:**
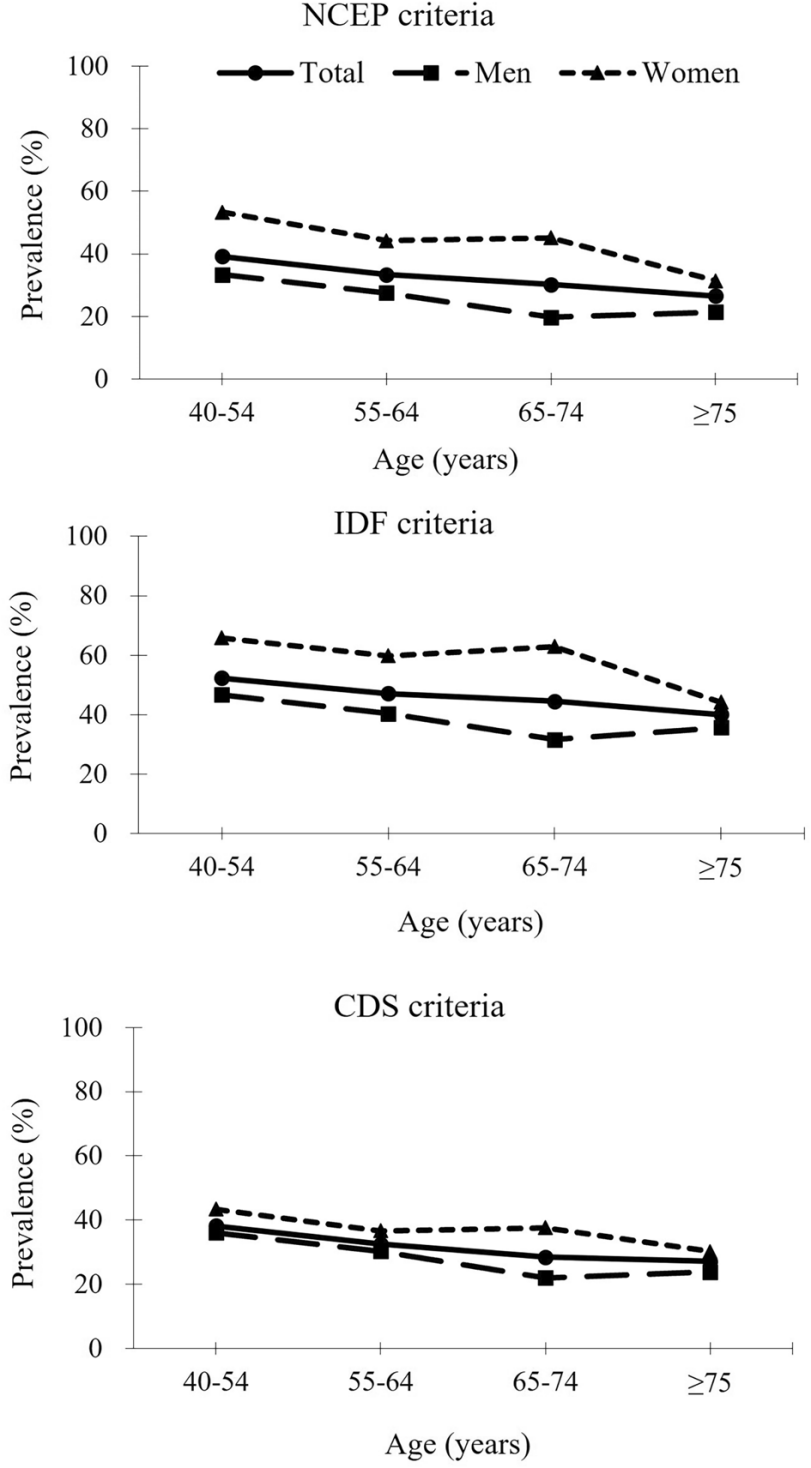
The age- and sex-specific prevalence (per 100 patients) of the metabolic syndrome defined by three sets of criteria in patients with acute ischemic stroke. *NCEP* National Cholesterol Education Program, *IDF* International Diabetes Federation, *CDS* Chinese Diabetes Society.

Figure 1 shows the age- and sex-specific prevalence of MetS defined by the three sets of criteria. The overall prevalence of MetS was 33.4% by NECP criteria, 47.2% by IDF criteria, and 32.5% by CDS criteria. For each criteria, women had a higher MetS prevalence than men across all age groups, and the sex difference disappeared after the age of 75 years. The prevalence of MetS decreased with age overall and for both men and women.

**Figure 2:**
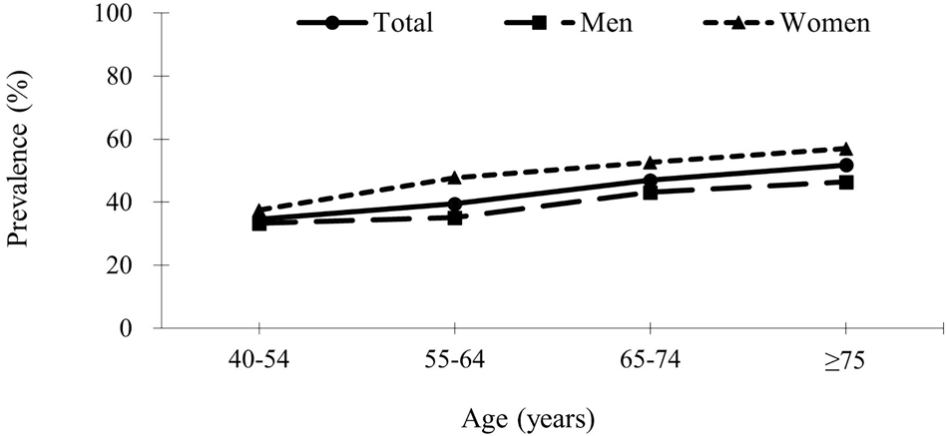
The age- and sex-specific prevalence (per 100 patients) of coronary heart disease in patients with acute ischemic stroke.

The overall prevalence of CHD among patients with ischemic stroke was 41.3% (48.1% in women; 37.4% in men, p < 0.05). The prevalence increased from 34.6% in those aged 40–54 years, 39.5% in those aged 55–64 years, 47.0% in those aged 65–74 years, to 51.8% in those aged ≥ 75 years, and the prevalence increased with age for both men and women (Fig. 2). The prevalence of CHD was higher in women than in men across all age groups.

In the total sample, high blood pressure, high serum triglycerides, and low HDL-C were significantly associated with CHD (OR ranged from 1.27 to 1.38), however, there was no significant association of abdominal obesity and high blood glucose with CHD (Table 3). The MetS defined by all three sets of criteria was associated with an increased likelihood of CHD, with the adjusted OR ranging from 1.27 to 1.44 (P < 0.05). When the analysis was stratified by sex, high blood pressure, high serum triglycerides, and MetS defined by IDF criteria was significantly associated with an increased likelihood of CHD in men, whereas among women, high serum triglycerides, low HDL-C, and MetS defined by all three sets of criteria were associated with CHD.

**Table 3:** The associations of metabolic syndrome and its individual components with coronary heart diseases in patients with acute ischemic stroke.

MetS and individual components^a^	Total sample (n = 1851)			Men (n = 1176)			Women (n = 675)		
	No. of patients	No. of CHD cases	OR (95% CI)^b^	No. of patients	No. of CHD cases	OR (95% CI)^b^	No. of patients	No. of CHD cases	OR (95% CI)^b^
**MetS components**									
**Abdominal obesity**									
No	1083	434	1.00 (Ref)	783	296	1.00 (Ref)	300	138	1.00 (Ref)
Yes	717	307	1.10 (0.89–1.35)	360	129	0.99 (0.75–1.30)	357	178	1.28 (0.93–1.77)
**High blood pressure**									
No	413	143	1.00 (Ref)	295	90	1.00 (Ref)	118	53	1.00 (Ref)
Yes	1419	616	1.36 (1.08–1.72)	868	347	1.51 (1.13–2.01)	551	269	1.14 (0.75–1.72)
**High blood glucose**									
No	993	391	1.00 (Ref)	674	245	1.00 (Ref)	319	146	1.00 (Ref)
Yes	844	366	1.14 (0.94–1.38)	492	190	1.15 (0.90–1.48)	352	176	1.14 (0.83–1.56)
**High triglycerides**									
No	1221	480	1.00 (Ref)	805	294	1.00 (Ref)	416	186	1.00 (Ref)
Yes	607	277	1.38 (1.12–1.70)	362	143	1.31 (1.00–1.72)	245	134	1.50 (1.08–2.09)
**Low HDL-C**									
No	855	323	1.00 (Ref)	642	231	1.00 (Ref)	213	92	1.00 (Ref)
Yes	957	427	1.27 (1.04–1.55)	515	202	1.18 (0.92–1.52)	442	225	1.49 (1.06–2.10)
**MetS by various criteria**									
**NCEP criteria**									
No	1232	481	1.00 (Ref)	860	317	1.00 (Ref)	372	164	1.00 (Ref)
Yes	619	284	1.27 (1.03–1.57)	316	123	1.16 (0.88–1.53)	303	161	1.46 (1.06–2.01)
**IDF criteria**									
No	978	362	1.00 (Ref)	709	250	1.00 (Ref)	269	112	1.00 (Ref)
Yes	873	403	1.44 (1.18–1.76)	467	190	1.36 (1.06–1.76)	406	213	1.62 (1.17–2.24)
**CDS criteria**									
No	1250	493	1.00 (Ref)	829	304	1.00 (Ref)	421	189	1.00 (Ref)
Yes	601	272	1.27 (1.03–1.57)	347	136	1.18 (0.90–1.55)	254	136	1.45 (1.05–2.01)

*MetS* Metabolic syndrome, *HDL-C* high-density lipoprotein cholesterol, *CHD* coronary heart disease, OR odds ratio, *CI* confidence interval, *NCEP* National Cholesterol Education Program, *IDF* International Diabetes Federation, *CDS* Chinese Diabetes Society.

^a^The individual components were defined according to the IDF criteria. The number of participants with missing values was 51 for abdominal obesity, 19 for high blood pressure, 14 for high blood glucose, 23 for high triglycerides, and 39 for low HDL-C.

^b^OR (95% CI) was derived from models that were controlled for age, education, smoking, alcohol drinking, physical inactivity, and dietary, and if applicable, for sex.

Furthermore, we categorized all participants into three groups according to the number of abnormal MetS components that were defined by each of the three MetS criteria, i.e., 0 (reference), 1–2, and ≥ 3 MetS components. In the total sample, compared to patients without abnormality in any of the five MetS components, having 1–2 and ≥ 3 abnormal MetS components was significantly associated with an increased likelihood of CHD (Table 4). There was no statistical interaction of MetS with sex on CHD. However, when the analysis was stratified by sex, the results showed that having 1–2 and ≥ 3 abnormal MetS components (vs. none) defined by all the three sets of criteria was significantly associated with an elevated likelihood of CHD in men, whereas in women only having ≥ 3 abnormal MetS components defined by the CDS criteria was significantly associated with an increased likelihood of CHD (Table 4).

**Table 4:** The associations between number of metabolic syndrome components and coronary heart diseases in patients with acute ischemic stroke.

No. of MetS components	Total sample (n = 1851)			Men (n = 1176)			Women (n = 675)		
	No. of patients	No. of CHD cases	OR (95% CI)^a^	No. of patients	No. of CHD cases	OR (95% CI)^a^	No. of patients	No. of CHD cases	OR (95% CI)^a^
**NCEP criteria**									
0	149	43	1.00 (Ref)	124	33	1.00 (Ref)	25	10	1.00 (Ref)
1–2	1083	438	1.66 (1.14–2.45)	736	284	1.84 (1.20–2.84)	347	154	1.30 (0.55–3.04)
≥ 3	619	284	2.01 (1.34–3.02)	316	123	1.98 (1.23–3.19)	303	161	1.87 (0.80–4.38)
**IDF criteria**									
0	114	31	1.00 (Ref)	96	25	1.00 (Ref)	18	6	1.00 (Ref)
1–2	864	331	1.66 (1.07–2.59)	613	225	1.72 (1.05–2.81)	251	106	1.68 (0.59–4.78)
≥ 3	873	403	2.28 (1.46–3.58)	467	190	2.20 (1.32–3.65)	406	213	2.63 (0.94–7.40)
**CDS criteria**									
0	164	47	1.00 (Ref)	120	33	1.00 (Ref)	44	14	1.00 (Ref)
1–2	1086	446	1.72 (1.19–2.48)	709	271	1.74 (1.12–2.69)	377	175	1.81 (0.91–3.61)
≥ 3	601	272	2.05 (1.39–3.02)	347	136	1.91 (1.19–3.07)	254	136	2.48 (1.22–5.03)

*MetS* Metabolic syndrome, *CHD* coronary heart disease, *OR* odds ratio, *CI* confidence interval, *NCEP* National Cholesterol Education Program, *IDF* International Diabetes Federation, *CDS* Chinese Diabetes Society.

^a^OR (95% CI) was derived from models that were controlled for age, sex, education, smoking, alcohol drinking, physical inactivity, and dietary, and if applicable, for sex.

## Discussion

### Summary of the main findings

MetS affects around one-third to nearly a half of patients with ischemic stroke, depending on the defining criteria for MetS, which ranged from 32.5% by CDS criteria and 33.4% by NECP criteria to 47.2% by IDF criteria. CHD was present in 41.3% of patients with ischemic stroke. The prevalence of both MetS and CHD was higher in women than in men, and the prevalence of CHD increased with age but the prevalence of MetS slightly decreased with age. In addition, MetS defined by all three sets of criteria was associated with an increased likelihood of CHD in patients with ischemic stroke. Notably, compared to patients without any of the five MetS components, having even 1–2 abnormal components was associated with a higher likelihood of CHD, especially in men.

### Compared with other studies

In our study, the prevalence of MetS defined by IDF criteria was 47.2% among patients with ischemic stroke, which was in line with the report from another study of stroke patients in China (51.3%)^24^. However, our prevalence of MetS was lower than that in Polish stroke patients (61.2%) based on the same criteria^25^. The difference might be partly due to a higher proportion of women in the Polish study than ours (57.6% vs. 36.5%) because women are more likely to have MetS than men. Indeed, we found that women had a higher prevalence of MetS than men across all age groups, which is in line with the reports of previous studies^2,25,26^. The sex difference might be primarily attributable to the higher levels of MetS components (e.g., waist circumference, systolic blood pressure, blood glucose, and triglycerides) in women than in men.

In addition, we found that the prevalence of MetS slightly decreased with age in both men and women. This was different from the previous studies, which reported an increasing prevalence with age in young or middle-aged people^27^ but a relatively stable prevalence with age in older adults^6,28^. The decreasing prevalence of MetS with age may be explained by the age-related metabolic and pathophysiological changes, due to the fact that the levels of some MetS components, e.g., waist circumference, diastolic blood pressure, and total cholesterol, may not increase with age, especially in very old age^29,30^.

Coronary heart disease is highly prevalent in patients with ischemic stroke and the risk of long-term fatal CHD following the onset of clinical stroke or TIA is increased^31^. In addition, the follow-up study of patients with ischemic stroke showed that new-onset cardiovascular complications (e.g., acute coronary syndrome, atrial fibrillation, and heart failure) diagnosed following an ischemic stroke were very common and that the newly diagnosed cardiovascular complications in patients with ischemic stroke were associated with an increased risk of recurrent stroke^16^. We found that the overall prevalence of CHD was 41% in patients with ischemic stroke, which can be supported by the previous studies showing that coronary atherosclerosis is present in around 45% of stroke patients^32,33^.

### The association between MetS and CHD

The meta-analysis revealed that MetS could double the risk of cardiovascular events in the general population^7^, but the risk of cardiovascular events associated with MetS in patients with stroke has not been well studied. Notably, whether the prognostic value of the MetS for cardiovascular events exceeds that of the sum of MetS individual components remains to be clarified^34^. This is releant because defining MetS as a binary entity (abnormalities in ≥ 3 vs. < 3 MetS components) might limit its power for predicting cardiovascular events^35^. Indeed, our study showed that having even 1–2 abnormal MetS components was associated with an increased likelihood of CHD. Thus, defining MetS as a binary entity could underestimate the association of clustering of cardiometabolic risk factors with risk of CHD. Our findings of the MetS-CHD associations among patients with ischemic stroke were consistent with those of our previous report from the general population of older adults living in the same area^6^. However, very few studies have investigated the association between MetS and risk of CHD in patients with clinical stroke, which limits the comparison of our results with the literature.

The underlying pathways linking MetS with CHD could be that MetS is associated with endothelial dysfunction and inflammation, which are key pathophysiologic features of atherosclerosis^36^. Atherosclerosis plays a key role in CHD through several critical processes in the pathogenesis of atherosclerosis (e.g., lipid accumulation, intimal thickening and fibrosis, vascular inflammation, remodeling, and plaque rupture or erosion)^37^. In addition, the ulceration of atherosclerotic plaques is very important in coronary occlusion^38^. Our analysis showed that having more components was linearly associated with an increased likelihood of CHD. This suggests that multiple individual MetS components may have an accumulative effect on the atherosclerotic process and increase the likelihood of CHD, which is in line with previous studies^39^.

### Strengths and limitations

This hospital-based study includes a relatively large sample of patients with first-ever ischemic stroke who were mostly from the rural areas (26.7% illiteracy) of southwest Shandong province, a less developed region compared to the eastern coastal areas. In addition, trained staff and clinicians performed comprehensive assessments on a range of health-related factors and health conditions, which allowed us to define MetS with different criteria and to control for multiple potential confounders. However, this study also has limitations. First, because the study participants were recruited from local two university hospitals (tertiary hospitals), the patient sample might not be representative of the patient population. This should be kept in mind when generalizing our study findings. Second, a considerable proportion (73.3%) of participants had missing data on waist circumference, and an imputed waist circumference based on age, sex, and body mass index was used instead. However, this approach has previously been validated in terms of correct classification of abdominal obesity (88.4%) and cardiometablic risk (91.5% in men and 99.5% in women)^23^, thus, any bias from the missing waist circumference is likely to be minimal. Finally, the cross-sectional nature of the study design does not allow us to determine the causal relationship between MetS and CHD, and the cross-sectional association might be subject to survival bias that usually leads to underestimation of the true associations.

## Conclusions

Our hospital-based study of patients with first-ever acute ischemic stroke suggested that MetS affects around one-third to a half of stroke patients, depending on the criteria used for defining MetS, and that CHD was present in over 40% of the patients. Furthermore, MetS is associated with CHD in patients with ischemic stroke, and even having 1–2 abnormal components without meeting the criteria for MetS is associated with an increased likelihood of CHD. Our study also revealed sex differences in the prevalences of CHD and MetS as well as in the associations of MetS and its some components with CHD in patients with ischemic stroke. These results, if confirmed in follow-up studies, will add further evidence to the notion that proper management of MetS and individual components may benefit cardiovascular health in patients with stroke.

## Data Availability

The datasets generated and/or analysed during the current study are not publicly available due to current regulations but are available from the corresponding author on reasonable request and approval from the data management committee.

## Abbreviations

MetS: Metabolic syndrome
NCEP: National Cholesterol Education Program
IDF: International Diabetes Federation
CDS: Chinese Diabetes Society
CHD: Coronary heart disease
TIA: Transient ischemic attack
STEPS: WHO STEPwise approach to Surveillance
SAGE: Study on Global Ageing and Adult Health
HDL-C: High-density lipoprotein cholesterol
OR: Odds ratio
CI: Confidence interval
